# Supplementary material for: Resveratrol Improves Endothelial Function by A PREP1-Mediated Pathway in Mouse Aortic Endothelial Cells
Source: Int J Mol Sci. 2023 Jul 25;24(15):11891. doi: 10.3390/ijms241511891 (PMC10419093; doi:10.3390/ijms241511891)
Supplement: Supplementary file 1 [file ijms-24-11891-s001.zip › ijms-2490517-supplementary.pdf]

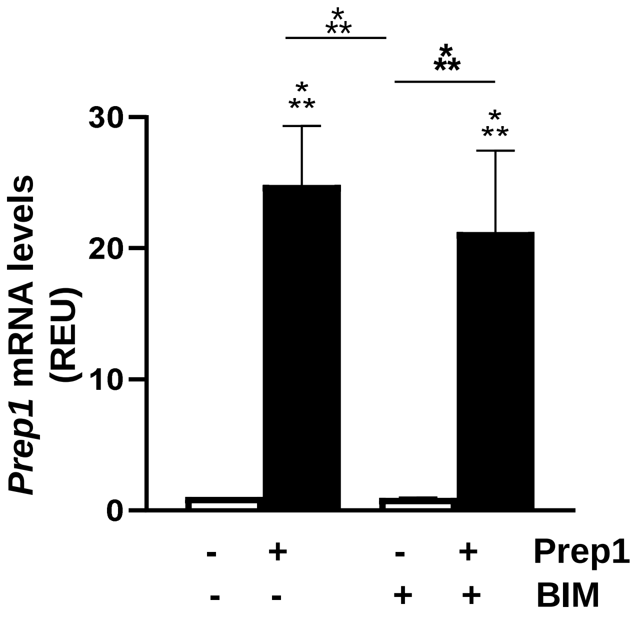

Supp. Figure

Supplemental Figure S1. Effect of PKCα inhibition on *Prep1* mRNA levels. CTRL and *Prep1* overexpressing MAEC cells were incubated with bisindolylmaleimide (BIM) at 100 nM concentration for 30 minutes. *Prep1* mRNA levels were evaluated by real-time RT-PCR analysis in CTRL and *Prep1* overexpressing MAEC cells. Data were normalized by the amount of *beta-actin*, used as internal control. Bars represent the mean ± SD of three independent experiments, each performed in triplicate. Asterisks denote statistical differences (***p<0.001).

ESM Table S1.

PRIMERS	SEQUENCES
Prep1	Forward: 5'-CCTGGGACAATTAGGATCCAGAACT-3'<br>Reverse: 5'-TCCTCTGTTGGGTAGGGATGCC-3'
TNFα	Forward: 5'-GATCGGTCCCCAAAGGGATG-3'<br>Reverse: 5'-CCACTTGGTGGTTTGTGAGTG-3'
IL-6	Forward: 5'- GCCAGAGTCCTTCAGAGAGA-3'<br>Reverse: 5'- GGTCTTGGTCCTTAGCCACT-3'
IL-1β	Forward: 5’-AACCTTTGACCTGGGCTGTC-3'<br>Reverse: 5’-CCTCATCCTGGAAGGTCCAC-3'
SOD2	Forward: 5’-CAAAGGAGAGTTGCTGGAGG-3’<br>Reverse: 5’-TCCTTGCAATGGGTCCTGAT-3'
ATF4	Forward: 5’-CTCATGGGGCCTTTAGGACG-3'<br>Reverse: 5’-GTGGTCACGTGATCCTACCG-3'
β-ACTIN	Forward: 5'-GGTGGGAATGGGTCAGAAGG-3'<br>Reverse: 5'-GTTGGCCTTAGGGTTCAGGG-3'

Primer sequences used in Real-time RT-PCR analysis.
